# Brain tissue- and region-specific abnormalities on volumetric MRI scans in 21 patients with Bardet-Biedl syndrome (BBS)

**DOI:** 10.1186/1471-2350-12-101

**Published:** 2011-07-27

**Authors:** Kim M Keppler-Noreuil, Catherine Blumhorst, Julie C Sapp, Danielle Brinckman, Jennifer Johnston, Peggy C Nopoulos, Leslie G Biesecker

**Affiliations:** 1 Department of Pediatrics, Division of Medical Genetics, The University of Iowa Children's Hospital, Iowa City, IA, 52242, USA; 2 Genetic Disease Research Branch, National Human Genome Research Institute, National Institutes of Health, Bethesda, MD, 20892-4472, USA; 3 Department of Psychiatry, The University of Iowa Children's Hospital, Iowa City, IA, 52242, USA

## Abstract

**Background:**

Bardet-Biedl syndrome (BBS) is a heterogeneous human disorder inherited in an autosomal recessive pattern, and characterized by the primary findings of obesity, polydactyly, hypogonadism, and learning and behavioural problems. BBS mouse models have a neuroanatomical phenotype consisting of third and lateral ventriculomegaly, thinning of the cerebral cortex, and reduction in the size of the corpus striatum and hippocampus. These abnormalities raise the question of whether humans with BBS have a characteristic morphologic brain phenotype. Further, although behavioral, developmental, neurological and motor defects have been noted in patients with BBS, to date, there are limited reports of brain findings in BBS. The present study represents the largest systematic evaluation for the presence of structural brain malformations and/or progressive changes, which may contribute to these functional problems.

**Methods:**

A case-control study of 21 patients, most aged 13-35 years, except for 2 patients aged 4 and 8 years, who were diagnosed with BBS by clinical criteria and genetic analysis of known BBS genes, and were evaluated by qualitative and volumetric brain MRI scans. Healthy controls were matched 3:1 by age, sex and race. Statistical analysis was performed using SAS language with SAS STAT procedures.

**Results:**

All 21 patients with BBS were found to have statistically significant region- and tissue-specific patterns of brain abnormalities. There was 1) normal intracranial volume; 2) reduced white matter in all regions of the brain, but most in the occipital region; 3) preserved gray matter volume, with increased cerebral cortex volume in only the occipital lobe; 4) reduced gray matter in the subcortical regions of the brain, including the caudate, putamen and thalamus, but not in the cerebellum; and 5) increased cerebrospinal fluid volume.

**Conclusions:**

There are distinct and characteristic abnormalities in tissue- and region- specific volumes of the brain in patients with BBS, which parallel the findings, described in BBS mutant mouse models. Some of these brain abnormalities may be progressive and associated with the reported neurological and behavioral problems. Further future correlation of these MRI scan findings with detailed neurologic and neuropsychological exams together with genotype data will provide better understanding of the pathophysiology of BBS.

## Background

Bardet-Biedl syndrome (BBS) is a heterogeneous, pleiotropic human disorder inherited in an autosomal recessive pattern, and characterized by the primary findings of obesity, polydactyly, hypogonadism, developmental delay or learning disabilities, and behavioural problems [1-3]. Most patients are affected by progressive retinopathy, renal disease, and susceptibility to diabetes mellitus, hypertension, and congenital cardiac malformations [1-3]. Currently, the diagnosis of BBS is made on the basis of clinical criteria [4]. Bardet-Biedl syndrome can be caused by mutations in as many as 16 genes [5-19]. The majority of proteins encoded by these genes have a role in the formation, stability and function of cilia [20]. In spite of the rapid progress in molecular understanding of BBS, it is only in recent years that extensive data on the natural history and molecular pathogenesis of this complex disorder have been reported [19].

Individuals with BBS may have cognitive and behavioral phenotypes. While the majority of patients do not have mental retardation, most cases have an intelligence quotient (IQ) in the low normal range [2]. Ataxia and impaired coordination evidenced by impaired tandem walking, slow repetitive supination/pronation of hands, and wide based gait were seen in a large patient series with BBS [4]. Of the 109 patients with BBS, of whom 40% had ataxia with poor coordination and 30% had gait abnormality, only 10 patients (9%) had brain imaging (CT or MRI), and there were no cerebellar or other brain abnormalities identified [4]. Moore et al. [3] suggested that the neurological abnormalities of paucity of facial muscle movement, ataxia and impaired coordination and mild hypertonia in all four limbs, suggest a central defect causing impaired coordination, which affects the brainstem, cerebellar, oculomotor and pyramidal tracts. Despite these and other reported neurological and behavioural problems in patients with BBS, there is limited documentation of neuroanatomical defects by brain imaging.

Analysis of various BBS mouse models confirms that defective ciliary development is the basis of some BBS phenotypes. These mice model features of the human phenotype and also have specific brain and neuroanatomical abnormalities [21-26]. The *Bbs1*^*M390R/M390R *^knockin mouse model had characteristic features of the human phenotype, including retinal degeneration, male infertility, obesity, and olfaction deficits, as did the *Bbs2, Bbs4, *and *Bbs6 *knockout mouse models [27]. Additionally, *Bbs1*^*M390R/M390R *^mice have a previously uncharacterized neuroanatomical phenotype of ventriculomegaly involving the third and lateral ventricles, thinning of the cerebral cortex, and reduction in the size of the corpus striatum and hippocampus [27].

A review of the literature for reported neuroanatomical defects in patients with BBS identifies 13 patients who have abnormal brain findings on neuroimaging [28-37]. These patients had documented major and minor findings to confirm a clinical diagnosis of BBS based upon the published clinical diagnostic criteria [28-37]. However, only three had testing for the BBS genes, one of the three had negative testing for 11 genes. Bauman et al. [29] described two patients with brain abnormalities of gyral atrophy, hydrocephalus, enlarged cerebral gyri, and hemispheric asymmetry. Baskin et al. [28] reported one patient with brain abnormalities of mega cisterna magna, and cerebellar vermis hypoplasia. Cerebellar atrophy was described in three separate patients [31,33,34]. Nonspecific cortical atrophy in one of five patients [37], and temporal and parietal lobe hypoplasia [30] were found in two patients with BBS. Soliman et al. [36] reported patients with empty sella with hypothalamic and pituitary abnormalities. Karmous-Benailly et al. [32] reported two fetuses with confirmed BBS mutations that were found prenatally to have brain abnormalities: Dandy-Walker malformation in one and agenesis of the corpus callosum in the other. A recent study of three patients reported evidence of cerebral cortical atrophy, cerebellar atrophy, lateral ventricle enlargement, temporal and parietal lobe hyperplasia, and microcephaly [35].

Case series of neuroanatomic abnormalities on clinical scans of patients can be useful in identifying patterns of brain pathology in disorders. However, a more powerful method of assessing abnormalities in brain structure is using research MRI scans to obtain quantitative measures of brain structure in groups of patients, comparing them to healthy controls. This technique provides accurate assessment of both global and regional brain structure. In the past 25 years, these types of studies in normal healthy populations have elucidated important information regarding developmental trajectories and sex differences in both brain structure and function [38]. Moreover, many studies have been conducted on patient populations with brain disorders, including a large number of developmental syndromes [39]. Although each patient population has its own unique signature of brain pathology, important global themes in quantitative MRI studies of developmental disorders include: abnormalities in tissue distribution between gray and white matter with several disorders showing gray matter or cortical 'enlargement' compared to controls [40-46]; 2) sex-specific morphologic changes [42,47,48] and 3) alterations in normal growth patterns of brain tissue over time [40].

To date, only one study has used quantitative MRI to evaluate patients with BBS. Baker et al. [49] recently reported on 10 patients with BBS compared to 10 controls and found structural abnormalities, including reduced total gray matter volume, but no total white matter or cerebrospinal volume changes; gray matter loss was in the anterior temporal lobes and in the medial orbitofrontal cortex and white matter volume loss in the right inferior longitudinal fasciculus [49]. They found regional brain volume loss in the hippocampus.

Based on the neurological and behavioural problems reported in patients with BBS, the paucity of brain imaging studies, and neuroanatomical abnormalities found in BBS mouse models, we conducted a qualitative *and *quantitative MRI study to assess brain morphology in patients with an established diagnosis of BBS.

## Methods

### Subjects

All 21 subjects were studied at the Clinical Research Center, National Institutes of Health (NIH) in Bethesda, MD, USA. The patients were included in the study after they gave (with their parents if they were minors) written informed consent (in accordance with the principles set forth in US Regulations 45CFR46, which governs human subjects research in the United States). This study protocol was approved by both the Institutional Review Board (IRB) of the National Human Genome Research Institute (NHGRI), at the National Institutes of Health (NIH), and by the IRB of the University of Iowa. The majority of the 21 patients ranged in age from 13 to 35 years, except for 2 patients aged 4 and 8 years, with 10 males and 11 females (see Table 1 for demographic information). Recruitment of subjects with BBS was through referrals from geneticists and genetic counselors, advertisements in genetics journals, and through patient support groups. The majority of the patients with BBS were selected by age (being >12 years) because of the ability to have a brain MRI scan without sedation. The diagnosis of BBS was made if the subject had either four of the six "primary" criteria or at least three of "primary" criteria, and 2 of the secondary criteria. Mutation analysis was performed in all patients for the known BBS genes. *MKS1 *and *CEP290 *were not analyzed in this cohort because causation has yet to be confirmed for these loci. A BBS gene locus was only considered causative if a patient had two mutations in the BBS gene. Whenever possible, both parents were enrolled for molecular testing for carrier status.

**Table 1:** Demographic Information on Patients and Controls

	**Patients with BBS (n = 21)**	**Healthy Controls (n = 63)**
Age in years (Mean, S.D.)	21.34, 8.41	18.57, 6.50
Age Range	4.91 - 35.75	7.08 - 35.00
% Male	47.62	47.62

Healthy control subjects were obtained from a database of children and adults who participated in brain imaging studies at the University of Iowa. This group of control subjects was chosen to be equivalent in age (mean and range) and sex composition relative to the BBS sample. All control subjects were excluded for the following: major medical, neurological, or psychiatric illness; previous head trauma; any metal implants.

### Image acquisition

For the control subjects, images were obtained at the University of Iowa on a combination of a 1.5 Tesla GE Signa MR scanner (n = 53 controls) or a 1.5 Tesla Siemens Avanto Scanner (n = 10 controls). All scans were obtained using a standard multi-modal protocol that including axial 3D volumetric spoiled gradient echo series (1 × 1 × 1.5 mm voxels) and a dual echo proton density/T2 (1 × 1 × 3 mm voxels) series. The sequences have been shown to be comparable across scanners in a previous study using images obtained on both scanners [50]. The BBS subjects were scanned at the National Institutes of Health using a Phillips 1.5T scanner. The parameters used for the acquisition protocol were designed to match the sequence obtained at Iowa. This procedure has been shown to produce comparable sequences in a previous large, multi-site MRI study [51].

### Post processing

All scans were processed through an automated procedure implemented in BRAINS [52], a family of tools designed for quantitative brain imaging, validated against manual tracing. The T1 image was reoriented by stepwise co-registration to a set of template images, centered on the anterior commissure, and re-sampled to 1 mm resolution. T2 and PD images were co-registered to the final T1 image, and all images were intensity normalized and inhomogeneity corrected. A discriminant tissue classification was then performed and a brain mask was created using an artificial neural network (ANN). Measures of gray matter, white matter, and CSF volumes were then completed using the standard Talairach method. Artificial neural networks were applied to each scan to measure subcortical structures, including caudate, putamen, and thalamus. These methods have been validated with pediatric samples [53]

We studied 21 affected individuals which were matched by age and sex 1:3 to normal control subjects. Each scan was evaluated qualitatively by a neuroradiologist for structural abnormalities.

For this quantitative analysis, a comprehensive examination of brain structure was done beginning with general measures of brain structure compared across groups including intracranial volume, total brain tissue volume, cerebellum volume, as well as volumes of tissue types (cerebrospinal fluid, gray matter, white matter). Intracranial volume (ICV) is the tissue and fluid within the calvarium and is a representation of the maximal brain growth obtained during development [54-56]. In addition, regional measures of the cerebrum (frontal, parietal, temporal, and occipital lobes) were also assessed, as well as volumes of subcortical structures (caudate, putamen, thalamus). Finally, sex-specific abnormalities in brain structure were evaluated.

### Statistical Analysis

All analyses were performed by using the SAS language with SAS STAT procedures. All brain measures were analyzed using the General Linear Models Procedure. Brain measures included general measures of intracranial volume (ICV) separated into total Cerebral Spinal Fluid (CSF) volume and total brain tissue volume. Total brain tissue was then separated into total white matter volume and total gray matter volume. Total CSF is separated into ventricle volume and 'surface' CSF (simple measure of total CSF minus ventricle volume). Regional measures included cerebellum tissue volume and cerebrum measures which were separated into the four cerebral lobes (frontal, parietal, temporal, occipital). Each cerebral lobe was broken down by gray matter and white matter volumes. Finally, measures of sub-cortical nuclei (caudate, putamen, and thalamus) were generated.

All measures are reported in cubic centimeters (CC). For the general measure of ICV, height and sex were used as covariates. However, to control for differences in overall head size, all measures were expressed and analyzed as a proportion of intracranial volume (ICV). Small structures (caudate, putamen, thalamus), are listed and labeled as ICV percentages to avoid excessive leading zeros. All possible interaction terms were entered into the model, but dropped if not significant. An alpha level of 0.05 (two-tailed) was used for significance tests.

## Results

There were 21 patients with an age range of 13 to 35 years, except for 2 patients aged 4 and 7 years. There were 10 males and 11 females. All had a clinical diagnosis of BBS made by diagnostic criteria [4]. Eighteen patients had two confirmed causative BBS gene mutations: seven patients with *BBS1 *mutations (not including one patient having only one mutation), six patients with *BBS10 *mutations, one patient with *BBS4 *mutations, one patient with *BBS5 *mutations, one patient with *MKKS *mutations, and one patient with *BBS12 *mutations. Three patients did not have identifiable mutations in *BBS1 - BBS12 *(we did not assess *TRIM32*). The control patient population had the same age range and male/female distribution.

Qualitative radiological interpretation of the MRI scans of the brain revealed that ten of 21 patients had apparently normal studies with no evidence of structural brain defects, while nine patients had significant findings. These included: two patients with the cerebellar tonsils ~2 mm below the foramen magnum (one of which had a likely pineal gland cyst); one with large pituitary gland and pars intermedia cyst of Rathke's cleft cyst; one patient with prominence of the sulci and cerebellar folia with normal ventricular system; one patient with prominent CSF space in the supratentorial part of the brain; one patient with colpocephaly, hypoplasia of the hypothalamus/thalamus, and subependymal nodule with Dandy-Walker variant; one patient with tiny foci of T2 scattered through the subcortical regions of the cerebrum and cerebellum; and two patients with mega cisterna magna and posterior fossa arachnoid cyst.

The analyses of the quantitative brain measures are shown in Table 2. For the general brain measures, the two groups did not differ on ICV. However, subjects with BBS had substantially smaller total brain tissue volumes and increased CSF volumes. Within the brain tissue, gray matter volume did not differ in the two groups; however white matter was substantially decreased in volume. Therefore, the decrement in total brain tissue volume in the BBS subjects appears to be accounted for primarily by white matter volume loss while gray matter volume is spared overall. Although both ventricle volume and surface CSF volume were both significantly expanded in the subjects with BBS, this was most robust in the surface CSF measure.

**Table 2:** Analyses of the Quantitative Brain Measures for Patients with BBS and Healthy Controls

	**Patients with BBS (n = 21)**		**Healthy Controls (n = 63)**				**Sex by Diagnostic Interaction**	
	
	**Adjusted Mean**	**Raw Mean**	**Adjusted Mean**	**Raw Mean**	F*	**p**	**F**	**p**
	
ICV (cm^3^)	1,399.05	1,394.12 (109.14)	1,396.66	1,393.70 (124.49)	0.01	0.924	2.48	0.119
Total Brain Tissue	0.913	1,269.60 (122.95)	0.948	1,321.96 (115.29)	23.07	<0.0001	0.97	0.327
Gray Matter	0.613	846.53 (88.11)	0.608	849.51 (63.43)	0.68	0.426	4.63	0.034
White Matter	0.299	423.06 (53.47)	0.339	472.45 (66.86)	42.37	<0.0001	0.54	0.463
CSF	0.086	124.45 (51.27)	0.051	71.73 (38.43)	23.07	<0.0001	0.97	0.327
Surface CSF	0.074	106.83 (48.07)	0.042	59.20 (35.03)	21.85	<0.0001	1.09	0.299
Ventricles	0.012	17.62 (7.09)	0.009	12.52 (9.21)	4.15	.0.450	0.00	0.952
Cerebral Cortex								
Frontal	0.197	267.92 (31.81)	0.194	268.99 (20.00)	0.55	0.461	5.31	0.023
Temporal	0.124	172.92 (17.86)	0.125	175.02 (14.24)	0.32	0.571	0.05	0.824
Parietal	0.104	143.85 (15.96)	0.105	147.36 (12.35)	0.36	0.551	0.73	0.395
Occipital	0.062	86.59 (9.73)	0.055	77.95 (7.88)	36.41	<0.0001	0.01	0.913
Cerebral White Matter								
Frontal	0.107	151.99 (20.70)	0.119	166.92 (24.59)	23.77	<0.0001	1.25	0.266
Temporal	0.042	60.02 (9.79)	0.049	68.60 (10.69)	39.36	<0.0001	0.00	0.963
Parietal	0.069	97.75 (13.99)	0.074	104.52 (15.67)	10.62	0.0016	0.04	0.844
Occipital	0.017	25.25 (5.75)	0.305	42.50 (9.31)	163.30	<0.0001	0.19	0.662
Cerebellum	0.105	145.77 (15.94)	0.103	143.42 (13.06)	1.93	0.168	0.49	0.487
Caudate	0.311	4.29 (0.58)	0.338	4.73 (0.69)	7.44	0.007	0.02	0.882
Putamen	0.776	10.68 (1.51)	0.834	11.63 (1.29)	7.34	0.008	1.53	0.220
Thalamus	0.846	11.79 (1.70)	0.885	12.32 (1.18)	4.77	0.032	2.22	0.139

*ANCOVA: controlling for age and sex; All brain measures (other than ICV) are expressed as volume/ICV ratios. For caudate, putamen and thalamus, measure is volume/ICV X100 (% of ICV).

In regional measures, the volume of the cerebral cortex in cases was not significantly different than controls in the frontal, temporal, or parietal lobes. However, the volume of the cortex in the occipital lobe was significantly increased in the patients with BBS compared to controls. In regard to white matter, all four cerebral lobes showed substantial decrements in volume in the patients with BBS compared to controls. This difference was largest in the occipital lobe.

The sub-cortical nuclei of caudate, putamen and thalamus were all decreased in volume in the patients with BBS compared to controls. However, the volume of the cerebellum was not different between the two groups.

### Sex effects

Although as a group, the subjects with BBS had no differences compared to controls in the gray matter measures, there were two measures that had significant sex by group interactions: total gray matter volume and frontal lobe gray matter volume. Table 3 shows these brain measures separated by sex. These analyses show that although the female subjects with BBS when compared to female controls were not different in these two measures, the male patients with BBS had significantly larger gray matter volumes (total and frontal lobe) compared to the male controls.

**Table 3:** Effects of Sex on Total Gray Matter and Frontal Lobe Gray Matter Volume*

	**Males with BBS**	**Control Males**	**t**	**P**	**Females with BBS**	**Control Females**	**t**	**p**
	
Total Gray Matter	0.622	0.606	2.09	0.039	0.604	0.611	0.958	0.346
Frontal Lobe Gray Matter	0.197	0.191	2.14	0.035	0.191	0.194	1.11	0.267

*Represent ratios of total gray matter and frontal lobe gray matter to total intracranial volume (ICV)

## Discussion

In this study, we present the results of cranial MRI imaging utilizing volumetric analyses of the CNS on 21 patients with the diagnosis of Bardet-Biedl syndrome confirmed by clinical diagnostic criteria and by genetic testing for the known BBS genes. This represents the largest case-control study of brain findings in BBS. We found that there are consistent brain abnormalities in patients with BBS: 1) normal intracranial volume; 2) reduced white matter in all regions of the brain, but most prominent in the occipital region; 3) preserved gray matter volume, with increased cerebral cortex volume in only the occipital lobe; 4) reduced gray matter in the subcortical regions of the brain, including the caudate, putamen and thalamus, but not in the cerebellum; 5) increased cerebrospinal fluid volume in both the surface of the brain and ventricles; and 6) there were sex differences: the gray matter volume in females with BBS did not differ from female controls, but that volume in males with BBS was significantly greater than male controls. The brain morphological abnormality in this study of substantially decreased white matter in the occipital lobe represents a unique observation that has not been described either in other patients with BBS or in subjects with isolated or nonsyndromic visual impairment.

Many of the current findings are consistent with the previous study by Baker et al. [49], including total brain volume decrement and global and occipito-temporal white matter volume decrement. However, there were some differences in the results of the two studies and we have extended the findings with novel quantitative findings. For the Baker study, the white matter volume did not reach statistical significance however was quantified as a nearly 5% reduction in global white matter volume. Here we show that this difference is statistically significant. In contrast to the current findings, the Baker study found both global and regional gray matter deficits. One possible reason for the difference in findings in the global gray matter is that the Baker sample was primarily female and our findings indicate that male BBS subjects tend to have enlarged cortical volumes. While the current study found that females with BBS had smaller volumes of total gray matter, it did not reach significance. Moreover, the regional deficits reported in the Baker study were small, focal areas of deficit (gyrus rectus, temporal poles and hippocampus) while the current study evaluated larger regions (cerebral lobes) in which small areas of deficit can remain undetected. They did not find cerebrospinal volume changes, or gray matter loss in the subcortical regions of the brain, which is found in this study and supported by findings in the BBS mouse models. The differences that were present between our studies may be attributed to several possible factors: 1) differences in quantitative MRI scan methodology; 2) their smaller sample size and control population; and 3) the lack of analysis of sex effects in their study.

The neuroanatomical abnormalities in the *Bbs1 *mutant, *Bbs*^*M390R/M390R*^, mice include ventriculomegaly involving the third and lateral ventricles, thinning of the cerebral cortex, and reduction in the size of the subcortical structures such as the corpus striatum and hippocampus [27]. In particular, the enlargement of the lateral ventricles is most severe, whereas the increased size of the third ventricle is less pronounced. The moderately enlarged lateral ventricles and a reduced corpus striatum are seen as early as postnatal week three. The pathology appears to be progressive based on greater enlargement of the lateral and third ventricles of 3.5 to 6-month-old mutant animals as assessed by coronal MRI. In addition to ventriculomegaly, the *Bbs1*^*M390R/M390R *^mice have thinning of the caudal half of the cerebral cortex and a reduction in the size of the corpus striatum and hippocampus. These neuroanatomical defects were also noted in the *Bbs2*^*-/-*^*, Bbs4*^*-/- *^*, Bbs6*^*-/- *^mice of comparable ages. There is no enlargement of the fourth ventricle in any of these mouse models. Neither the current study nor the previous study by Baker et al. [49] found evidence of ventriculomegaly in humans with BBS. However, we did indeed find reduction in the volume of the striatum, suggesting that the mouse models the human in this respect.

Intracranial volume or ICV is a measure of maximal brain growth; it is the volume of the internal skull cavity and is determined by brain growth, which peaks around the age of 12 years [54-56]. The fact that the subjects with BBS had normal ICV suggests that their maximal point of brain growth was no different than that of the controls. However, their total tissue volume was substantially decreased suggesting that some brain process after peak growth had decreased the volume of their brain. Because the previous tissue composition was not measured, we cannot assess the processes that led to decreased tissue. Possibilities include a lack of myelination (normally myelin is laid down until the 3^rd^, even 4^th ^decade of life) or demyelination; another possibility is excess pruning which happens normally in adolescence, but that would suggest that the cortex started out enlarged and was normal size when the patients were scanned. Another possibility is atrophy with frank cell death. The CSF space enlargement is also supportive of the notion that there is fluid where tissue once was - supporting the hypothesis that there was loss of cerebral tissue. There was no evidence of obstructive hydrocephalus due to aqueductal stenosis based upon clinical findings of absent normal fourth ventricle size and normocephaly. Consistent with the findings of progressive tissue loss after development, the *Bbs1 *mutant mice brains showed progressive pathology based on the greater enlargement of the lateral and third ventricles of 3.5- to 6-month-old mutant animals as assessed by coronal MRI [27].

The regional structural abnormalities in this study are important to highlight as well, to consider the potential functional correlations. One of the most significant findings was that of cortical enlargement and white matter reduction in the occipital lobe. This abnormality did not have a sex effect and thus was evident in the whole group. This finding suggests a functional correlate since most subjects in the study had progressive retinal degeneration with vision loss. The observation of white matter loss in the occipital lobe in other forms of retinopathy has not been described in the literature from our review. As one of the major functions of the occipital cortex is visual processing, we hypothesize that patients with BBS have a compensatory increase in occipital cortex because of progressive visual loss. There is little available published data in both the mouse models and human studies regarding brain morphology and visual loss, in particular regional cortical brain analyses. However, there are two reports, which describe analyses of the visual cortex in humans with early- and late-onset blindness. This study found that the bilateral visual cortices of the blind were thicker than those sighted controls, which may support the hypothesis that sensory experience is necessary for appropriate regression and remodeling of neuronal processes and that synaptic regression might be a major determinant of macroscopic anatomical features like cortical thickness. It has been found that pruning of the synapses depends on visual input. Therefore, these studies support our finding of increase in the volume of the occipital cortex since all our patients had evidence of rod-cone dystrophy [[57],58]. Another finding of the entire group with no sex effect is in the reduction of the striatum which could be related to the motor abnormalities seen in patients with BBS such as slow supination/pronation and poor tandem walking. This may be particularly relevant to the fact that the cerebellum in both the current study and in the Baker study was found to be structurally normal, suggesting that these motor abnormalities may not be related to a primary cerebellum dysfunction.

Finally, the regional abnormality of enlarged frontal lobe volume in the male subjects with BBS is an interesting and somewhat unexpected finding. The pattern of a developmental syndrome having cortical enlargement is not uncommon, being reported in a variety of disorders including oral clefting [42], Van der Woude Syndrome [43], autism [44], neurofibromatosis [41], Attention Deficit Disorder [45], and Williams Syndrome [46]. In oral clefting, the cortical enlargement was seen only in males and was directly related to cognitive and behavioral abnormalities of inattention and hyperactivity [57]. Although sex-specific abnormalities in cognition and behavior in BBS has not been reported, studies in the future should evaluate the patterns of cognitive and behavioral abnormalities and relate them to structural abnormalities such as seen here in the current study.

The current study has several limitations that should be considered. First, there were several different scanners used across the various groups (both BBS and controls). Although validation studies have shown the measures produced from these scanners to be comparable, a potential confounder would be created if indeed measures differed between scanners. Also, the age range of both groups varied widely. Although the control group had sex and age appropriate subjects, evaluation of age and developmental effects can't be adequately addressed, given the small sample size of the BBS subjects and the non-normal distribution of ages (in the BBS sample, over half of the sample was 20 years of age and over and only 2 subjects were below the age of 12). Future directions would include expanding the study to include more subjects in the younger age ranges (below 18 years).

## Conclusions

We conducted a case-control study comparing the brain findings in patients with Bardet-Biedl syndrome to a matched control population. There were distinct and characteristic abnormalities in tissue- and region- specific volumes of the brain, which correspond to several findings, described in BBS mutant mouse models. We identified novel and different quantitative brain abnormalities, extending those findings described in the Baker study [49]. We show that there is a statistically significant difference in the white matter volume in patients with BBS, with cortical enlargement in the occipital lobe. There was preserved total gray matter volume in our sample, including the cerebellar region, which was controlled for not only age differences but also sex differences. Contrasting with the Baker study, we found decreased cerebrospinal volume and gray matter loss in the subcortical regions of the brain, which mirrors the neuroanatomical findings in the BBS mouse models. In addition, the results of this study indicate that some of these brain abnormalities may be progressive indicated by preserved intracranial volume between the controls and BBS patients accompanied by substantially smaller total brain tissue volumes, in addition to associated neurological and behavioral problems reported in patients with BBS. While the use of quantitative MRI scan is useful on a research basis, these data do not suggest that MRIs are clinically indicated for BBS. Future studies to correlate these MRI findings with detailed neurologic and neuropsychological examinations and genotype data will provide better understanding of the pathophysiology of BBS.

## Competing interests

All authors have nothing to disclose.

## Authors' contributions

KKN has made contributions to conception and design or acquisition of data and wrote the manuscript. CB, JCS, and DB have made contributions to acquisition of data. JJ performed and interpreted the genetic analysis of BBS genes. PN has made contributions to conception and design, performed statistical analyses and interpretation of data, and wrote portions of the manuscript. LGB has made contributions to conception, design, and revised the manuscript critically for important intellectual content. All authors read and approved the final manuscript.

## Pre-publication history

The pre-publication history for this paper can be accessed here:

http://www.biomedcentral.com/1471-2350/12/101/prepub
